# Initiation of DNA Replication: The Genomic Context

**DOI:** 10.1371/journal.pbio.0020184

**Published:** 2004-06-15

Every time a cell divides, it must first duplicate its entire genome. Barring the occasional error, the daughter cells inherit identical copies of the parent cell's genome. With a typical human cell containing almost 9 feet of DNA made of 3 billion base pairs crammed into a nucleus about 5 microns (.0002 inches) in diameter, that's no small feat. To accomplish the job, cells engage specialized teams of protein machines, each performing different tasks during the various stages of DNA replication: initiation, duplication, quality control, and repair. Much of what we know about the molecular mechanisms of DNA replication comes from studies of bacteria. In the bacterial genome, which consists of several million base pairs, replication begins at a single site, spanning about 100 base pairs. The regulation and mechanisms of replication, even in the compact bacterial genome, are so complex that 51 years after Watson and Crick reported that the structure of DNA “immediately suggests” a mechanism for its replication, biologists are still working out the details and regulation of that mechanism.[Fig pbio-0020184-g001]

**Figure pbio-0020184-g001:**
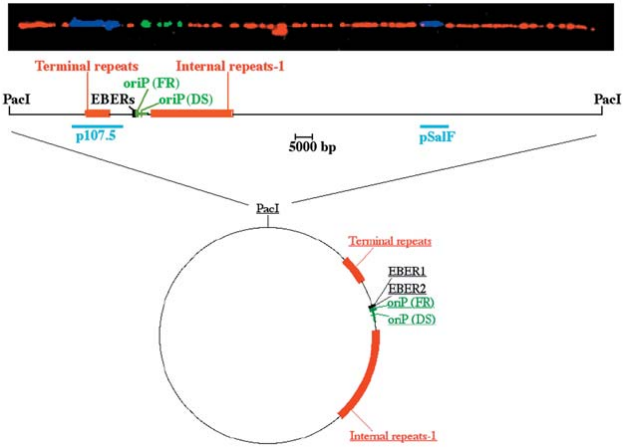
Linearized EBV episome imaged by fluorescent microscopy and aligned with the corresponding genomic map

Before duplication, aptly named initiator proteins bind to DNA at replication initiation sites and break the bonds holding the complementary base pairs together, separating the double helix locally into single strands and creating two Y-shaped junctions at either end called replication forks. At each replication fork, a complex of proteins continues the business of unzipping the DNA and using the exposed single strands as templates to generate complementary daughter strands. What controls when and how individual initiation sites are activated in mammalian cells has remained obscure. Is initiation restricted to specific sites? Do specific DNA sequences control initiation events locally? Examining individual molecules of fluorescently labeled replicating DNA, Paolo Norio and Carl Schildkraut report that initiation events are not controlled by individual initiation sites but occur throughout the genome. And the activation of these sites appears to depend on what's happening at the genomic level.

Using a novel technique called single molecule analysis of replicated DNA (SMARD), Norio and Schildkraut use the Epstein Barr virus (EBV) in human B cells as a model system for studying DNA replication. During the latent stage of infection, the EBV genome exists as an episome—a circular piece of extrachromosomal DNA. It replicates only once per cell cycle, during the DNA synthesis stage, and uses its host's replication machinery to do so. Using nucleotide analogs that can be detected by immunofluorescence (since the analogs attract antibodies that are fluorescently labeled), the researchers can determine the position, direction, and density of the replication forks, and then determine how replication starts, progresses, and terminates.

Norio and Schildkraut studied replication using two strains of the EBV virus grown in human B cells, their natural target. Previous studies, which had largely focused on the activity of individual initiation sites, had suggested that different EBV strains vary in how initiation sites are activated and that specific initiation sites or regions likely regulate replication. Looking at larger genomic regions, Norio and Schildkraut found something different: not only do initiation sites occur throughout the genomes, but their activity “differs dramatically” in the two EBV strains and even within a strain. Differences were seen in the order of initiation site activation, in the direction of replication fork movement, and in the speed of duplication in different parts of the genome. While the two largely similar viral genomes do show some genetic differences, the authors dismiss the idea that these local differences could explain the observed variations in replication control. It's more likely, they conclude, that epigenetic modifications (such as changes in chromatin structure) produce the differences in the order and frequency of activation of initiation sites across genomic regions.

It seems that initiation events are not restricted to specific genomic areas, and experimentally induced loss of individual initiation sites does not significantly affect EBV genome replication (because other sites take up the slack). This redundancy provides flexibility in determining which sites are activated. Since the EBV genome uses human replication machinery to duplicate its genome, these findings likely apply to DNA replication in mammalian cells as well. The very survival of the cell—and the health of the organism it inhabits—depends upon the faithful replication of the genome. Using processes that operate at the genomic level may afford cells the means to manage an unwieldy genome, and perhaps, more importantly, guarantee their genes safe passage to the next generation.

